# GNN-DRL-Based Intelligent Routing and Resource Allocation Algorithms for Multi-Layer Wireless Mesh Network

**DOI:** 10.3390/s26041170

**Published:** 2026-02-11

**Authors:** Lei Xu, Shu Han, Wei Fu, Ziran Zhu, Jing Wu, Xiaorong Zhu

**Affiliations:** Nanjing Yuanneng Power Engineering Co., Ltd., Nanjing 210000, China

**Keywords:** wireless mesh network, graph neural network, routing algorithm, multi-agent deep reinforcement learning, resource optimization

## Abstract

This research introduces a new intelligent routing and resource allocation algorithm called Graph Sample and Aggregate-Multi-Agent Proximal Policy Optimization (GraphSAGE-MAPPO), which targets dynamic wireless mesh networks like those present in emergency communications. Aiming to address the emergency communication scenario where the network topology changes dynamically and the introduction of Artificial Intelligence (AI) model training services leads to more diverse user services and more dynamic node resource capabilities, a three-dimensional mesh network intelligent routing and resource allocation algorithm, GraphSAGE-MAPPO, based on Graph Neural Networks (GNN) combined with Deep Reinforcement Learning (DRL), is proposed. During the training process, the algorithm first uses GNN as a network feature extraction module to extract the resource capabilities and link status indicators of the nodes, thereby generating a hidden feature vector representation for each backbone mesh node; then, the feature vectors of each node are combined with the arrival service flow as the state input of the distributed multi-agent DRL model, supporting efficient routing and resource allocation decisions for service flows with different user Quality of Service (QoS) requirements. Simulation results show that in the face of dynamically changing network environments and user needs, the GraphSAGE-MAPPO algorithm proposed in this thesis can flexibly adjust routing strategies to better meet the QoS requirements of various services and has good generalization performance for network topology and resource changes. These results demonstrate that the algorithm has good flexibility and scalability in large-scale, real-world wireless mesh network environments.

## 1. Introduction

Wireless Mesh networks, with their distributed architecture and flexible resource scheduling capabilities, demonstrate advantages in terms of comprehensive coverage and differentiated services in scenarios such as emergency response, smart cities, and industrial Internet of Things (IoT). In recent years, as the environment of wireless Mesh networks has become increasingly complex and the diversity of service traffic has expanded, routing optimization and efficient resource allocation have emerged as significant research hotspots. A key research question is how to achieve unified, adaptive, and user satisfaction-oriented multidimensional resource scheduling and routing optimization for multiple types of services in a dynamic Mesh network environment.

Compared to traditional routing algorithms based on fixed rules, DRL can dynamically optimize path selection and resource allocation strategies for diverse traffic flows by online learning of the spatiotemporal characteristics of network traffic and the patterns of service demands. Ref. [1] presents a comprehensive performance comparison of routing forwarding rules generated by supervised learning and reinforcement learning, indicating that supervised learning, which lacks interaction with the environment, struggles to model effectively, whereas reinforcement learning achieves optimization objectives for improved network performance through interaction with the environment to learn routing forwarding strategies. Addressing the energy efficiency optimization problem in wireless sensor networks, Ref. [2] proposes a data aggregation-aware routing algorithm based on Q-learning mechanisms, employing a dynamically designed reward function to achieve balanced energy consumption distribution; experimental results demonstrate that this approach can extend the network’s lifespan by 23%. To tackle the issue of insufficient representational capability of reinforcement learning in complex network scenarios, which can lead to slow or ineffective learning processes, Ref. [3] introduces an intelligent routing algorithm based on Deep Q-Networks, utilizing neural networks to learn optimal routing strategies. The findings indicate that the DRL-based intelligent routing algorithm can capture network states more accurately and make superior routing decisions compared to traditional methods and reinforcement learning-based strategies [4]. Furthermore, to address the challenge of high retraining costs in highly dynamic environments such as vehicular networks, Ref. [5] proposes a computing and communication cost-aware service migration scheme enabled by Transfer Reinforcement Learning. By leveraging knowledge transfer, this method significantly reduces the training time required to obtain optimal policies compared to standard schemes, ensuring better satisfaction of delay requirements.

However, due to the distributed characteristics of wireless Mesh networks, the action space and state space of the DRL model significantly increase as the network scale expands and concurrent traffic surges, leading to a slow convergence of the training process or even difficulties in convergence. Consequently, centralized DRL models are challenging to apply in large-scale networks. Ref. [6] proposes a multi-agent Actor-Critic architecture reinforcement learning algorithm in the context of wireless Mesh networks to balance end-to-end delay and channel efficiency, demonstrating its effectiveness. Ref. [7] introduces an online routing algorithm based on multi-agent DRL, which generates routes in a hop-by-hop manner and employs deep neural networks to learn traffic flow scheduling strategies under different QoS requirements, thereby meeting the QoS demands of various network services.

As research progresses, scholars are increasingly recognizing that wireless networks essentially consist of a set of nodes and their interconnections, which collectively form a complex graph structure rich in feature information. Recognizing this structural characteristic, early works such as Ref. [8] have successfully utilized graph theory-based clustering schemes for resource allocation and interference coordination in heterogeneous cellular networks. This approach demonstrated that explicitly modeling the neighborhood relationships between cells and users can significantly improve spectral efficiency. Existing intelligent algorithms based on DRL struggle to accurately model and process this graph structure. This difficulty arises because current methods [5,6] primarily employ standard neural networks, such as fully connected neural networks and convolutional neural networks, to construct agent models. These networks cannot accurately extract and fully utilize high-dimensional data, such as the features of network nodes and links. Consequently, it becomes challenging to generalize the trained models to different network topologies [9], resulting in suboptimal performance of DRL agents when evaluated in previously unseen topological environments.

Fortunately, as a neural network model specifically designed for graph structures, Graph Neural Networks (GNNs) can derive feature vector representations for each node through multiple rounds of information interaction and aggregation with neighboring nodes, demonstrating a strong generalization capability to changes in network structure. To further enhance the representation capability of GNNs in radio resource management, Ref. [10] introduces a general edge-update empowered GNN architecture (ENGNN). By extending the standard node-update mechanism to explicitly handle variables defined on edges, this method achieves superior scalability and generalization across varying network sizes and interference levels. Similarly, Ref. [11] proposes an offline reinforcement learning algorithm based on GNN for radio resource allocation in Ultra-Reliable Low Latency Communication (URLLC) services within 5G networks, exhibiting stable convergence and meeting the QoS requirements for URLLC. Ref. [12] integrates Deep Q-Networks (DQN) with GNNs to address the association problem between users and base stations, resulting in a 10% improvement in utility compared to traditional DQN methods. Ref. [13] introduces a technique that combines GNN and DRL to tackle resource allocation issues in cellular vehicular networks, ensuring a high success rate for Vehicle-to-Vehicle (V2V) communications while minimizing interference with vehicle and infrastructure links; the results indicate that the proposed algorithm effectively enhances the decision-making quality of agents with a minimal increase in computational load. Ref. [14] reformulates the cooperative navigation problem of multi-drone systems’ underground base station communication coverage as a Markov game, proposing a GNN-based Multi-Agent Proximal Policy Optimization (GMAPPO) algorithm that effectively integrates extracted latent features related to drones with their state space, thereby improving navigation safety. Ref. [15] addresses the challenges posed by a large number of satellites, rapid changes in network topology, and diverse traffic demands in low Earth orbit satellite inter-routing, designing a GNN and DQN integrated solution framework aimed at minimizing the end-to-end average latency across the network; GNNs are employed to learn the complex relationships between satellite nodes, while DQNs are utilized to formulate routing decisions that adapt to the characteristics of the satellite network. Ref. [16] presents an online routing optimization algorithm, G-Routing, which utilizes GNN in conjunction with DRL; by modeling and understanding the relationships between various network features, it predicts trends in service latency and throughput, allowing agents to learn optimal paths that adapt to environmental changes, with simulation results indicating faster convergence and improved performance across various metrics. Ref. [17] proposes a Dense GNN-DRL model to address the issues of low bandwidth utilization and insufficient model generalization capabilities in smart grids, further enhancing the feature aggregation capacity of graphs through the combination of GNN and recurrent neural networks.

The literature above conducts an in-depth exploration of network resource allocation or routing optimization for GNN-assisted agent decision-making across various scenarios; however, most of the studies focus primarily on a single type of service or performance metric.

Therefore, in response to the increasing and dynamically changing user demands and network topologies in wireless Mesh networks, this paper employs GNN in conjunction with DRL techniques for resource allocation and routing optimization in wireless Mesh networks. Initially, the inductive learning capability of GraphSAGE was utilized to extract the hidden feature vectors of nodes and edges within the wireless Mesh network. Subsequently, these hidden feature vectors, along with the locally observed traffic arrival conditions at the agent nodes, are used as inputs for the multi-agent Proximal Policy Optimization (PPO) algorithm. A centralized training and distributed execution architecture is adopted, with each backbone Mesh node functioning as an independent agent executing the algorithm, thereby enabling efficient decision-making in traffic routing and resource scheduling.

The main contributions of this paper are as follows:

- A node feature aggregation algorithm based on GraphSAGE is proposed, which utilizes the inductive GraphSAGE GNN model to extract node resource capabilities and link state indicators, generating hidden feature vectors for each node. This approach possesses inductive learning capabilities, enhancing the model’s generalization ability and enabling it to adapt to the frequent changes of nodes in large-scale dynamic Mesh networks. The algorithm is designed with a mechanism of global centralized training combined with feature vector distribution. During the training phase, it uniformly aggregates features and constructs globally consistent node representations, effectively improving the accuracy and consistency of network state representations. Additionally, it incorporates service flow conditions as input for a distributed multi-agent DRL model, thereby facilitating efficient and adaptive routing and resource allocation decisions.
- The topology-adaptive GraphSAGE-MAPPO algorithm is proposed, integrating the inductive bias of GNN with DRL to fundamentally address the “input dimension mismatch” challenge in dynamic emergency communication Mesh networks. The algorithm leverages the inductive GraphSAGE model to extract node feature vectors invariant to network scale, and characterizes the state, action, and reward functions of the DRL through a multi-agent Markov process. The action space encompasses discrete routing node selection and bandwidth resource allocation, as well as continuous allocation of computational and storage resources. The Multi-Agent Proximal Policy Optimization (MAPPO) algorithm is employed as the DRL implementation, retaining the “clipping” proximal update strategy of Proximal Policy Optimization (PPO) to constrain policy updates, thereby avoiding policy mutations and ensuring training stability, which is particularly suitable for the rapidly changing network conditions characteristic of Mesh network scenarios. Furthermore, MAPPO effectively accommodates a mixed action space by integrating optimization objectives for both discrete and continuous actions, thereby supporting diverse user QoS requirements.
- Through simulation validation, the proposed algorithm demonstrates the ability to flexibly adjust routing strategies, outperforming other algorithms in terms of average delay, packet loss rate, network throughput, and the accuracy of AI service training. Particularly in dynamic scenarios such as network node failures, the trained model exhibits exceptional transferability, effectively adapting to the dynamic changes in network topology and resource capacities, while maintaining robust generalization performance in complex network environments and diverse user demands.

## 2. System Model

### 2.1. Network Model

**Figure 1:**
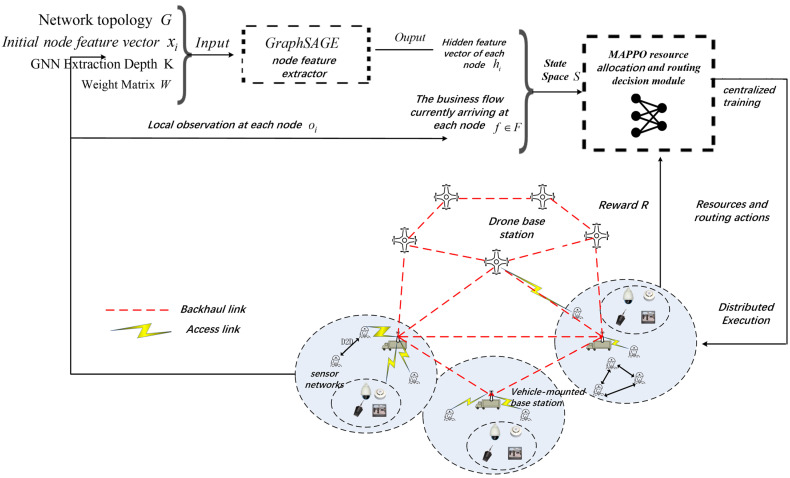
Architecture Diagram of Stereoscopic Wireless Mesh Network Based on GNN-DRL.

This paper considers the emergency communication scenario of vacant three-dimensional wireless Mesh networks, as illustrated in Figure 1. The network comprises various types of entity nodes, including mobile terminal nodes, unmanned aerial vehicle (UAV) aerial base station nodes, sensor nodes, and ground vehicle-mounted base station nodes, with the number of nodes and topology dynamically varying within a certain range. To explicitly model these dynamics, we assume that the mobile nodes follow the Random Waypoint mobility model, where nodes move towards randomly selected destinations with velocities uniformly distributed within a specific range, resulting in a time-varying network topology. Terminal nodes and various sensor nodes generate traditional communication services such as voice, video, and data collection and uploading, as well as novel AI services like small model training, necessitating the network to provide real-time dynamic services that meet QoS requirements. Considering that energy efficiency is typically a constraint in such scenarios, we clarify the scope of this study: for the purpose of focusing on routing logic under high mobility, we assume the nodes possess sufficient battery capacity for the duration of the mission.

A directed graph represents the network scenario $G=\left(N,L\right)$, where N denotes the set of nodes and L represents the set of links. Nodes such as drone base stations and vehicular base stations provide forwarding and computational services for user traffic flows. All nodes share a total bandwidth of *B* from the spectrum resources, which are divided into $K_{B}$ unit subchannels. Base station nodes can allocate one or more subchannel bandwidth resources to traffic flows based on the service demands of the traffic and their own service capabilities. For the sake of clarity in subsequent representations, the i node in the network is denoted as $n_{i}$, with user nodes labeled as $n_{UE}$, and base station nodes as $n_{BS}$. The number of user nodes is $N_{UE}$, and the number of base station nodes is $N_{BS}$. The network link set L consists of the set of inter-base station links $L_{s}$ and the set of user access links $L_{u}$, expressed as $L=L_{s}\cup L_{u}$. The link set dynamically changes with the addition or removal of nodes and variations in the network topology.

In the process of wireless transmission, the link channel gain between the nodes $n_{u}$ and $n_{v}$ within the communication range is denoted as $g_{uv}$. At any given moment in the network, multiple data flows requiring service may coexist. Due to the shared utilization of the same spectral resources among various Mesh nodes, there is a potential for transmission interference among service flows allocated to the same sub-channel bandwidth. Furthermore, a single node or link may simultaneously serve multiple services at a given time, necessitating consideration of resource allocation efficiency.

Let the transmission power of the node $n_{u}$ be $p_{u}$, then the signal-to-noise ratio (SNR) for data received by the node $n_{v}$ can be expressed as:

(1) $$SINR_{uv}=\frac{g_{uv}p_{u}}{\sum_{k\in N\backslash \{i\}}g_{kv}p_{k}+\sigma_{u}^{2}}$$

The term $\sigma_{u}^{2}$ represents the power of the additive white Gaussian noise (AWGN) at node $n_{u}$. Consequently, the link capacity between the two nodes is given by:

(2) $$C_{uv}=B_{l_{uv}}\log_{2}(1+SINR_{uv})$$

Here, $B_{l_{uv}}$ denotes the bandwidth allocated to the link $l_{ij}$. The achievable throughput of a multi-hop path is constrained by the minimum capacity link, which is referred to as the bottleneck link. Consequently, the bottleneck transmission rate for the traffic flow f is expressed as:

(3) $$C_{min}^{f}=\underset{l\in path_{f}}{min}C_{l}$$

In this context, $path_{f}$ denotes the end-to-end service path of the service flow. The transmission delay from the user to the base station refers to the time required for data packets to be transmitted between the user and the access base station. The transmission delay of the service flow f from user $n_{UE}$ to base station $n_{BS}$ is expressed as:

(4) $$T_{tran}^{n_{UE},n_{BS}}=\frac{data_{n_{UE},n_{BS}}^{f}}{C_{n_{UE},n_{BS}}}d(n_{UE},n_{BS})$$

Among them, $data_{n_{UE},n_{BS}}^{f}$ represents the data volume of the service flow f, $d(n_{UE},n_{BS})$ denotes the distance between user $n_{UE}$ and access base station $n_{BS}$, and $C_{n_{UE},n_{BS}}$ indicates the link transmission rate.

The computational processing delay refers to the time spent by the base station processing the service data packets after receiving them. Let the computational processing capacity allocated by the base station and $n_{BS}$ to user $n_{UE}$ for service f be denoted as $c_{n_{BS}}^{n_{UE}}$. Consequently, the base station processing delay for the user $n_{UE}$ accessing the base station $n_{BS}$ is given by $\frac{data_{n_{UE},n_{BS}}^{f}}{c_{n_{BS}}^{n_{UE}}}$ $\bar{delay_{l_{uv}}}$.

In the network backhaul, the transmission delay between the base stations $n_{BS}^{u}$ and the next-hop base station $n_{BS}^{v}$ can be expressed as:

(5) $$T_{tran}^{n_{BS}^{u},n_{BS}^{v}}=\frac{data_{n_{BS}^{u},n_{BS}^{v}}}{C_{n_{BS}^{u},n_{BS}^{v}}}d(n_{BS}^{u},n_{BS}^{v})$$

The end-to-end delay of the service flow is the sum of the base station’s computation processing delay and the path transmission delay.

At the same time, considering the packet loss rate of data transmission, the packet loss rate of the link between two nodes is defined as the ratio of the number of lost packets, denoted as $N_{uv}^{loss}$ to the total number of packets sent, denoted as $N_{uv}^{send}$ Over a statistical time period. This can be expressed as:

(6) $$P_{uv}^{loss}=(\frac{N_{uv}^{loss}}{N_{uv}^{send}})*100\%$$

The end-to-end packet loss rate of the service f is represented by the cumulative packet loss condition across each segment of the service path $path_{f}$:

(7) $$P_{path_{f}}^{loss}=1-\prod_{l\in path_{f}}\left(1-P_{l}^{loss}\right)$$

### 2.2. Service Model

When a user node $n_{i}$ initiates a service request, if the end-to-end destination node for its service is $n_{j}$, a service flow $f_{ij}$ will be established upon successful network access, defined as $f_{ij}=\{type_{f_{ij}},data_{f_{ij}},C_{f_{ij}}^{demand},req_{f_{ij}}\}$. Here, $type_{f_{ij}}$ denotes the type of service, with a value of 1 indicating traditional communication services such as voice, video, and sensor data uploads, where the QoS metrics include transmission rate, latency, and packet loss rate; a value of 0 signifies novel AI services, which in this paper refers to Deep Neural Network (DNN) model inference and training tasks, with QoS metrics encompassing not only the indicators as mentioned above but also training accuracy. $data_{f_{ij}}$ represents the volume of service data, $C_{f_{ij}}^{demand}$ indicates the computational resources required to complete the task. The set of QoS metric requirements for the service flow is represented as $req_{f_{ij}}=\{delay_{f_{ij}}^{\max},rate_{f_{ij}}^{\min},lossrate_{f_{ij}}^{\max},accuracy_{f_{ij}}^{\min}\}$, where if a particular metric is not of concern, its corresponding position is assigned a value of 0. The collection of all service flows within the network is defined as F, where $f_{ij}\in F$. To characterize the dynamic nature of network traffic, we assume that the generation of these service requests follows a Poisson process to simulate random arrival intervals, and the source-destination pairs $\left(n_{i},n_{j}\right)$ are randomly selected from the network to ensure diverse traffic patterns.

For traditional communication services, the computational demands are relatively low. During the routing decision-making process, nodes that can meet the computational requirements are selected for processing, while the remaining nodes in the route serve merely as transmission relays. However, it is still essential to ensure the QoS metrics during the transmission process.

For AI services, a complete task process encompasses the inference task data and the Deep Neural Network (DNN) model required for training [18]. The specific training process is not the focus of this paper; rather, the emphasis lies on how to allocate network resources for these training tasks and select appropriate routing. Therefore, this paper assumes that the required DNN model has already been pre-trained. When the base station node first receives a task training request, the model will be deployed to that node from the cloud server, and no further redeployment will be necessary. It is assumed that the tasks are non-divisible; during the routing process of the service flow, the network attempts to dispatch the task. If the task remains processable at the destination node, it will be considered a service failure.

The AI service involves metrics related to model accuracy, with the accuracy of trained Deep Neural Network (DNN) models and data quality being two crucial factors influencing the training accuracy of AI tasks [9,10]. During the training process, due to resource constraints, different algorithms with varying compression rates may be selected. Compressed training models exhibit smaller sizes and consume fewer resources; however, this compression also results in a decrease in model accuracy. On the other hand, data quality is affected by factors such as channel conditions and transmission distance during the transmission process. Poor quality of data likewise leads to a reduction in training accuracy. This paper does not focus on the compression of DNN models; rather, it primarily considers how to reasonably select the training model’s precision based on network resources, link conditions, and service requirements, as well as how to enhance data transmission quality to improve overall training accuracy.

Define the data transmission quality of each link as a binary variable $\vartheta_{uv}$ where

(8) $$\vartheta_{uv}=\left\{\begin{array}{l} 1,\;P_{uv}^{loss}<P_{uv}^{loss\_th}\\ 0.95,\;P_{uv}^{loss}\geq P_{uv}^{loss\_th} \end{array}\right.$$

Inspired by the literature [18], we define the parameter representation of the pre-trained DNN model as $M_{z}=\{P_{z},Q_{z},S_{z}\},\;z\in \{0,1\}$, where $P_{z}$, $Q_{z}$ and $S_{z}$ represent the model’s inference accuracy, computational power requirements, and memory footprint, respectively. This paper provides two levels of DNN models for AI applications, where z=0 denotes a lightweight model, and z=1 indicates a full-scale model. The specific numerical relationships between model magnitude, model accuracy, computational power requirements, and memory size are presented in Table 1.

The training accuracy ultimately achieved by the AI system is equal to the transmission quality of each link during the transmission process multiplied by the training accuracy of the pre-trained DNN model. The training accuracy ultimately achieved by the AI system is equal to the transmission quality of each link during the transmission process multiplied by the training accuracy of the pre-trained DNN model, which can be expressed as: $accuracy=\vartheta_{uv}\cdot P_{z}$ where accuracy represents the final training accuracy, $\vartheta_{uv}$ denotes the transmission quality defined in Equation (8), and $P_{z}$ is the training accuracy of the pre-trained DNN model as listed in Table 1.

## 3. Problem Modeling

In wireless mesh networks, when performing resource allocation and routing optimization for traditional communication services and AI services, it is essential to simultaneously consider conventional QoS metrics, including transmission rate, latency, and packet loss rate, alongside the training accuracy metrics specific to AI services. Given the varying QoS requirements of different services, employing a singular metric as the standard for measuring service quality fails to ensure fairness and efficient resource utilization.

Therefore, this paper establishes a unified utility function for different types of services, normalizing the latency, rate, packet loss rate, and training accuracy of the traffic flow, and representing the four demand indicator vectors of the service flow as {delay,rate,lossrate,accuracy}. Different service flows exhibit differentiated requirements for various indicators, and the normalization formula for these indicators is defined as follows:

(9) $$delay_{norm}=\left\{\begin{array}{l} \frac{delay_{max}-delay}{delay_{max}},\;if\;delay<delay_{max}\\ 0,\;else \end{array}\right.$$

(10) $$rate_{norm}=\left\{\begin{array}{l} 0,\;if\;rate<rate_{min}\\ \frac{rate-rate_{min}}{rate_{max}-rate_{min}},\;if\;rate_{min}\leq rate\leq rate_{max}\\ 1,\;else \end{array}\right.$$

(11) $$lossrate_{norm}=\left\{\begin{array}{l} \frac{lossrate_{max}-lossrate}{lossrate_{max}},\;if\;lossrate<lossrate_{max}\\ 0,\;else \end{array}\right.$$

(12) $$accuracy_{norm}=\left\{\begin{array}{l} \frac{accuracy-accuracy_{min}}{accuracy_{min}},\;if\;accuracy>accuracy_{min}\\ 0,\;else \end{array}\right.$$

In this context, $rate_{norm}$, $lossrate_{norm}$ and $accuracy_{norm}$ represent the normalized values of delay, transmission rate, packet loss rate, and training accuracy, respectively. It is important to note that different services have varying threshold requirements for the metrics as mentioned above.

Let $I_{weight}(\tau )=\{I_{delay}(\tau ),I_{rate}(\tau ),I_{lossrate}(\tau ),I_{accuracy}(\tau )\}$ be the indicators for evaluating the performance weights of the traffic type τ, with the sum equal to 1. The rationale behind this weighted design is to address the multi-objective optimization nature of heterogeneous services. The weighting parameters $I_{weight}(\tau )$ are not arbitrary but are selected based on the specific QoS priorities of the traffic type τ. For instance, for delay-sensitive services, a higher value is assigned to $I_{delay}$, whereas for AI inference tasks, $I_{accuracy}$ is prioritized. This parameter selection ensures that the reward signal accurately reflects the distinct Service Level Agreements (SLAs) required by different applications.

(13) $$\begin{array}{l} Utility(\tau )=\frac{1}{\sum I_{weight}}(I_{delay}(\tau )delay_{norm}+I_{rate}(\tau )rate_{norm}\\ +I_{lossrate}(\tau )lossrate_{norm}+I_{accuracy}(\tau )accuracy_{norm}) \end{array}$$

The average service utility function for all user service types in the network is represented as $\frac{\sum_{\tau }Utility(\tau )}{\left|\tau \right|}$.

Define the binary variable $\chi_{f_{ij}}^{n}$ to indicate whether the task is processed at node n; it takes the value of 1 if true, and 0 otherwise. Define $b_{f_{ij}}^{l_{uv}}$ as the bandwidth allocated to the service flow $f_{ij}$ on link $l_{uv}$, measured in MHz. Define $c_{f_{ij}}^{n}$ as the computational resource capacity allocated to $f_{ij}$ at node n, measured in GHz. Define $s_{f_{ij}}^{n}$ as the amount of storage resources allocated to $f_{ij}$ at node n, measured in MB. Define $\varphi_{f_{ij}}^{z}$ to indicate whether the AI service flow $f_{ij}$ selects a compressed DNN model, where z denotes a lightweight model, and otherwise indicates a full-scale model. Additionally, for the sake of clarity, define the binary variable $\psi_{f_{ij}}^{l_{uv}}$ to represent whether the service flow $f_{ij}$ traverses link $l_{uv}$ in the physical topology; it takes the value of 1 if true, and 0 otherwise. However, $\psi_{f_{ij}}^{l_{uv}}$ is determined by $\chi_{f_{ij}}^{n}$ during the decision-making process and does not require separate handling.

The objective of this paper is to maximize the average service utility of users across the entire network, namely,

(14) $$\underset{\chi_{f_{ij}}^{n},b_{f_{ij}}^{l_{uv}},c_{f_{ij}}^{n},s_{f_{ij}}^{n},\varphi_{f_{ij}}^{z}}{\max}\frac{\sum_{\tau }Utility(\tau )}{\left|\tau \right|}$$

The constraints that need to be satisfied are:

(15) $$\sum_{v:l_{uv}\in L}\psi_{f_{ij}}^{l_{uv}}-\sum_{k:l_{ku}\in L}\psi_{f_{ij}}^{l_{ku}}=\left\{\begin{array}{l} 1,\;i=str_{f_{ij}}\\ -1,\;i=des_{f_{ij}},\;\forall f_{ij}\in \\ 0,\;otherwise \end{array}\right.F$$

(16) $$\sum_{f_{ij}\in F}\psi_{f_{ij}}^{l_{uv}}b_{f_{ij}}^{l_{uv}}\leq B_{l_{uv}},\;\forall l_{uv}\in L,\;f_{ij}\in F$$

(17) $$\sum_{f_{ij}\in F}\chi_{f_{ij}}^{n}c_{f_{ij}}^{n}\leq C_{n},\;\forall n\in N,\;f_{ij}\in F$$

(18) $$\sum_{f_{ij}\in F}\chi_{f_{ij}}^{n}s_{f_{ij}}^{n}\leq S_{n},\;\forall n\in N,\;f_{ij}\in F$$

(19) $$delay_{f_{ij}}\leq delay_{f_{ij}}^{max},\;\forall f_{ij}\in F$$

(20) $$rate_{f_{ij}}\geq rate_{f_{ij}}^{min},\;\forall f_{ij}\in F$$

(21) $$lossrate_{f_{ij}}\leq lossrate_{f_{ij}}^{max},\;\forall f_{ij}\in F$$

(22) $$accuracy_{f_{ij}}\geq accuracy_{f_{ij}}^{min},\;\forall f_{ij}\in F$$

Among them, constraint (15) indicates that for each service flow $f_{ij}$, the routing from the source node $str_{f_{ij}}$ to the destination node $des_{f_{ij}}$ must satisfy the flow conservation principle; constraint (16) imposes a limitation on the link bandwidth capacity, stating that the sum of the bandwidth consumed by the service flows traversing a particular link must not exceed the link’s bandwidth capacity; constraint (17) pertains to the computational resource limitations at nodes, specifying that the total amount of computational resources utilized by the computational tasks undertaken by a node must not exceed the total computational resources available to that node; constraint (18) addresses the storage resource limitations at nodes, indicating that the current storage of DNN models and processing data at a node must not surpass the total storage resources of that node; constraint (19–22) sets forth requirements regarding the latency, packet loss rate, minimum transmission rate, and training accuracy for AI services related to the demands of the service flows.

## 4. Design of Routing and Resource Allocation Algorithms for Mesh Networks Based on GNN-DRL

As user demands, network topologies, and resource statuses continually evolve, the application of traditional heuristic algorithms or mathematical optimization methods may result in failure to promptly release resources from users who have completed their service. Upon the completion of a single execution of the algorithm, only a static strategy tailored to the current set of users and network conditions can be obtained. However, when nodes are added or removed from the network, changes occur in user request types, and link quality and network available resources fluctuate over time, the original static strategy is unable to adapt swiftly to the new network environment. This leads to a reduction in resource utilization efficiency and poses challenges in meeting the continuously dynamic task demands of users in future emergency scenarios.

To this end, it is essential to seek an intelligent strategy capable of adapting to the dynamic changes in network topology and service requirements. GNNs, as neural network models specifically designed for graph structures with strong generalization capabilities, can directly extract information such as node adjacency relationships, link performance metrics, and available resources from Mesh networks, thereby forming high-dimensional hidden feature vectors for the nodes. Furthermore, by leveraging DRL techniques, multi-agent systems can make real-time decisions in a distributed environment based on the features extracted by GNN. The actions taken can autonomously adjust according to the learned strategies in response to updates in network topology, resource conditions, and variations in service demands. Consequently, this paper will design an intelligent algorithm based on GNN-DRL to address the proposed optimization of routing and resource allocation in Mesh networks.

This paper proposes a distributed Graph Neural Network based Multi-Agent Deep Reinforcement Learning (GNN–MADRL) routing and resource allocation algorithm in which each base station functions as an agent. Each base station is modeled as an agent, and the decision-making process encompasses network feature representation based on GraphSAGE and collaborative routing decisions based on MAPPO. The following sections provide a detailed introduction to the algorithm design.

### 4.1. Node Feature Aggregation Algorithm Based on GraphSAGE

GraphSAGE is an inductive GNN model that aggregates information from the current node and its adjacent nodes to form feature vectors, enabling the representation of any node through an aggregation function. In large-scale dynamic mesh networks, GraphSAGE allows each node to learn hidden feature vectors by relying solely on a subgraph of its immediate neighborhood, thereby exhibiting generalization capabilities. However, distributed subgraph training may lead to inconsistencies in the hidden vector information among nodes. Consequently, this paper adopts a global centralized training approach combined with the distribution of hidden vectors for node feature aggregation.

The model input includes: (1) Network topology information $G=\left(N,L\right)$; (2) The initial feature vectors of each node $x_{n_{i}}=\{pos_{n_{i}},B_{n_{i}},C_{n_{i}},S_{n_{i}}\}$, representing the position, currently allocable bandwidth, current computational resources, and current storage capacity of each node; (3) The initial feature vectors of each link $x_{l_{uv}}=\{\bar{throughput_{l_{uv}}},\bar{lossrate_{l_{uv}}},\bar{delay_{l_{uv}}}\}$, where $\bar{throughput_{l_{uv}}}$ denotes the historical average throughput of the link, $\bar{lossrate_{l_{uv}}}$ represents the historical average packet loss rate of the link, $\bar{delay_{l_{uv}}}$ indicates the historical average transmission delay of the link, which are sampled and collected from training sample data; (4) Network depth K, referring to the number of layers in the feature aggregation process through which feature propagation occurs. (5) Weight matrix W, which is continuously updated during the training process by calculating the loss gradient through backpropagation.

The model output consists of the hidden feature vectors of each node $h_{i}^{\left(K\right)}$.

**Figure 2:**
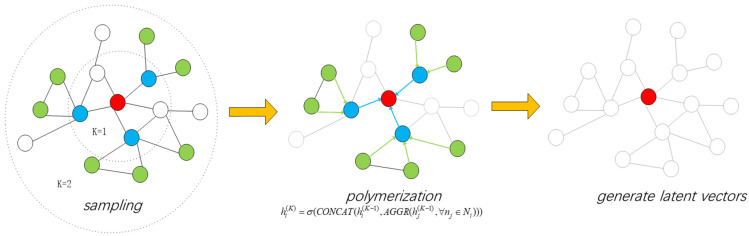
Schematic Diagram of Node Feature Vector Generation by GraphSAGE.

As illustrated in Figure 2, assuming the target node is $n_{i}$, the process of aggregating and updating the features of the neighboring nodes using the GraphSAGE model is as follows:

1. Neighbor node sampling

To balance network scale and computational efficiency, a partial sampling strategy is employed for aggregating neighbor information. Specifically, for each node $n_{i}$, a fixed number s of neighbors is randomly sampled from its adjacent node set $Adj_{i}$ for feature aggregation. If the number of neighbors is less than s, sampling is conducted with replacement to ensure that the total number of sampled adjacent nodes reaches s; if the number of neighbors exceeds s, random sampling without replacement is utilized, selecting only s nodes from all neighbors to control computational load. The number of samples per layer may vary; Figure 2 demonstrates a sampling process with a network depth of 2, where the number of sample nodes in the first layer is 3, and the number of samples in the second layer is 7.

In this study, due to the potential for each node to connect to multiple links, we aim to fully leverage the link feature information while avoiding an excessively large input space for the GNN. For each node $n_{i}$, its initial feature vector $x_{n_{i}}=\{pos_{n_{i}},B_{n_{i}},C_{n_{i}},S_{n_{i}}\}$ is extended in length and redefined as $x_{n_{i}}=\{pos_{n_{i}},B_{n_{i}},C_{n_{i}},S_{n_{i}},0,0,0\}$. When a node $n_{j}$ is selected during sampling, the feature vector of link $l_{uv}$, represented as $x_{l_{uv}}=\{\bar{throughput_{l_{uv}}},\bar{lossrate_{l_{uv}}},\bar{delay_{l_{uv}}}\}$, is appended to the last three zeros of the feature vector of $n_{j}$. This approach enables effective collection of both node and link features, allowing subsequent DRL processes to make targeted decisions based on the QoS requirements of the service flow.

2. Feature aggregation of adjacent nodes

For each layer of the network, the feature vector of each node is generated by the previous layer of the network, and each layer employs a method of sampling adjacent nodes to aggregate the features of neighboring nodes. The process of generating the hidden feature vector $h_{i}^{1}$ in the first layer of the network is represented as follows:

(23) $$h_{i}^{1}=\sigma \left(W^{1}\cdot \mathrm{CONCAT}\left(h_{i}^{0},\mathrm{AGGR}\left(\left\{h_{j}^{0}:\forall n_{j}\in {\mathrm{Adj}}_{i}\right\}\right)\right)\right)$$

In this context, W represents the parameter matrix that is progressively learned during the training process, while σ(·) denotes the nonlinear activation function. By aggregating the initial features of the s neighboring nodes of node $n_{i}$, denoted as $h_{j}^{0},\;\forall n_{j}\in Adj_{i}$, the aggregated features for the neighbors of $n_{i}$ are obtained as $h_{Adj_{i}}^{1}$. Subsequently, the aggregated features $h_{Adj_{i}}^{1}$ are linearly combined with the initial features $h_{i}^{0}$ of node $n_{i}$ using a residual connection. This is then multiplied by the parameter matrix to yield the hidden vector representation $h_{i}^{1}$ of the target node $n_{i}$ in the first layer of the network after undergoing a nonlinear transformation.

The mapping process of the intermediate hidden layers is like the above expression, represented as:

(24) $$h_{i}^{k}=\sigma \left(W^{k}\cdot \mathrm{CONCAT}\left(h_{i}^{k-1},\mathrm{AGGR}\left(h_{j}^{k-1},\forall n_{j}\in {\mathrm{Adj}}_{i}\right)\right)\right)$$

The output of the final hidden layer is the ultimate hidden feature vector of the node $n_{i}$, denoted as $h_{i}^{\left(K\right)}$. Consequently, the GraphSAGE model can produce the final hidden feature vectors for all nodes.

3. Parameter Learning

In the GraphSAGE model, the parameter matrix W defines the nonlinear mapping relationship between node features and neighborhood aggregation features. During the offline pre-training phase, the network parameters of the DRL model remain fixed, allowing the weight parameter matrix W of the GraphSAGE model to be adjustable. Since the overall objective is to maximize the cumulative reward of the routing task, we define R(W) as the objective loss function parameterized by the weight matrix W. Consequently, the stochastic gradient descent algorithm is employed to update the weights. The optimization process is represented as:

(25) $$W^{t}=W^{t-1}-\gamma \nabla_{W}R(W)$$

In this context, $W^{t}$ denotes the model weight parameters at step *t*, γ represents the learning rate, and $\nabla_{W}R(W)$ indicates the gradient with respect to the parameter W. Through continuous iterations, the model progressively acquires node neighbor feature representations that are conducive to routing decisions. Upon completion of the pre-training phase, the obtained W can be applied to new network nodes to generate hidden vectors with enhanced discriminative power and generalization capability, thereby providing support for the subsequent policy optimization of the DRL routing model.

The implementation of the feature extraction algorithm based on GraphSAGE is illustrated in the pseudocode presented in Algorithm 1.
**Algorithm 1.** Feature Extraction Algorithm Based on GraphSAGE.**Input:** directed graph G, initial node feature vector $\left\{x_{0},x_{1},\ldots ,x_{N_{s}-1}\right\}$, sample size s, sampling depth K, weight matrix $W^{k}$, nonlinear activation function σ(·), aggregation function Mean**Output:** Hidden feature vector of each node $\left\{{h_{i}}^{\left(K\right)},\forall n_{i}\in N_{s}\right\}$1:initial state of each node in the network $h_{i}^{0}\leftarrow x_{i},\forall n_{i}\in N_{s}$
2:**for** *k* = 1, 2, …, *K*3:**for** $\forall n_{i}\in N_{s}$4:Sample *s* neighbor nodes of $n_{i}$ and aggregate them using a mean aggregator:$h_{Adj(n_{i})}^{k}\leftarrow Mean(\{h_{v}^{k-1},v\in Adj(n_{i})\})$5:Concatenate the aggregated neighbor features $h_{Adj(n_{i})}^{k}$ with the node features $h_{n_{i}}^{k-1}$:$h_{n_{i}}^{k}\leftarrow \sigma (W^{k}\cdot CONCAT(h_{n_{i}}^{k-1},h_{Adj(n_{i})}^{k}))$6:**end**7:Normalize the result using a norm $h_{n_{i}}^{k}\leftarrow h_{n_{i}}^{k}/||h_{n_{i}}^{k}|{|}_{2}$8:**end**9:Obtain the final hidden feature vector of the node $h_{i}\leftarrow {h_{i}}^{\left(K\right)},\forall n_{i}\in N_{s}$

The computational complexity of the proposed feature extraction algorithm is analyzed as follows. Let $N_{s}$ be the number of nodes, K the search depth, s the neighbor sample size, and d the feature dimension.

Time Complexity: The algorithm iterates through K layers and $N_{s}$ nodes. In each iteration, it performs neighbor aggregation (O(s·d)) and feature transformation using matrix multiplication $\left(O(d^{2})\right)$. Thus, the total time complexity is $O(K\cdot N_{s}\cdot (s\cdot d+d^{2}))$. Since K, s, d are constants, the complexity is linear with respect to the number of nodes, i.e., $O(N_{s})$, ensuring low processing latency for real-time applications.

Space Complexity: The space requirement consists of storing the node feature matrices $\left(O(N_{s}\cdot d)\right)$ and the weight parameters $\left(O(K\cdot d^{2})\right)$. The overall space complexity is $O(N_{s}\cdot d+K\cdot d^{2})$, which is efficient for deployment on UAVs with limited memory resources.

### 4.2. Representation of Mesh Node Proxies Based on Markov Models

For a given service request from the source node $n_{i}$ to the destination node $n_{j}$, the routing generation process can be modeled as a series of Markov decision processes involving various agents along the service path from $n_{i}$ to $n_{j}$. The resource allocation and routing generation process of agent i deployed at node $n_{i}$ can be represented by a triplet $<S_{i},A_{i},R_{i}>$, where $S_{i}$ denotes the local observation state space of agent i, $A_{i}$ represents the action space of agent i and $R_{i}$ signifies the reward received by agent i upon taking action $a_{i}$. The following section will provide a detailed design of the key components of the DRL agent, using agent i deployed at $n_{i}$ as an example.

1. The state space $S_{i}$

The agent i formulates the state input $S_{i}$ for the DRL algorithm by integrating the node hidden feature vector $h_{i}$ obtained from the sampling and aggregation process based on the GraphSAGE model, along with the current incoming service flow information. This is expressed as $S_{i}=\{h_{i},f_{ij}\in F\}$, where $h_{i}$ represents the hidden feature vector of node extracted by the pre-trained GraphSAGE encoder, which explicitly captures local topological structures, dynamic link states, and available nodal resources. Crucially, to facilitate effective encoding, the continuous variables within these states are first normalized to [0, 1] via the mapping functions defined in Equations (9)–(12). Additionally, $f_{ij}=\{type_{f_{ij}},data_{f_{ij}},C_{f_{ij}}^{demand},req_{f_{ij}}\}$ denotes the service flow characteristics, including type, data volume, computational requirements, and various QoS demands.

2. Action space $A_{i}$

To explicitly structure the decision variables, the action space $A_{i}$ is defined as a joint tuple $A_{i}=(a_{resource},a_{route})$, comprising two distinct decision components:

Resource allocation actions $a_{resource}$: Allocate the bandwidth resources of $b_{f_{ij}}^{l_{uv}}$ to the traffic flow $f_{ij}$ utilizing the link $l_{uv}$. It is important to note that the bandwidth size of each unit sub-channel in this paper is denoted as $\frac{W}{K}$, and the allocated $b_{f_{ij}}^{l_{uv}}$ must be an integer multiple of $\frac{W}{K}$. Allocate computational resources $c_{f_{ij}}^{n}$ to the traffic flow $f_{ij}$ executing computational tasks at the node n; allocate storage resources $s_{f_{ij}}^{n}$ to the traffic flow $f_{ij}$ passing through the node n.

Routing Action $a_{route}$: The selection of the next hop node $n^{\prime }$ is determined by the adjacent relationships of the current node, resulting in a set of optional nodes. Consequently, as defined above, the composite action $\left(a_{route},a_{resource}\right)$ allows the agent to jointly optimize topology selection and resource distribution. To mitigate the potential for an excessively large action space that could hinder algorithm convergence, the implementation of the algorithm adopts a “demand-based allocation, best-effort” strategy when the current node is handling a single flow. In cases where multiple flows require processing, priority is given to servicing the flow that yields a greater service utility.

3. The reward function $R_{i}$

To achieve the optimization objective of maximizing the average service utility for users across the entire network, it is essential to design a rational instantaneous reward for the agent at each decision-making step. The reward function is defined as the utility value resulting from the execution of actions on the service flow $f_{ij}$, with negative rewards imposed in the event of constraint violations. Furthermore, to mitigate unnecessary exploration, the reward is set to a single-step maximum value of 1 when the current node is the endpoint of the flow. Specifically, the reward function $r_{i}^{t}$ obtained by agent I, after taking a routing action at time slot t is calculated based on various performance metrics, including latency, throughput, packet loss rate, and training accuracy, and is expressed as follows:

(26) $$r_{i}^{t}=\left\{\begin{array}{ll} -1, & \mathrm{if}\;\exists k\in Q,k_{\mathrm{norm}}=0\\ 1, & \mathrm{if}\;n_{i}=des_{f}\\ Utility(f), & \mathrm{otherwise} \end{array}\right.$$

where Q={delay,rate,lossrate,accuracy} represents the set of QoS metrics. The definition of each condition is as follows: Constraint Violation (−1): If any normalized QoS metric becomes 0, a penalty of −1 is imposed to strictly discourage actions that violate service agreements. Target Reached (1): If the current node $n_{i}$ is the destination $des_{f}$, a maximum reward of 1 is granted to encourage flow completion. Step Forwarding (Utility(f)): For intermediate steps where constraints are satisfied, the reward equals the weighted link utility Utility(f), encouraging the selection of high-quality links that maximize the overall path utility.

The objective of Agent i is to maximize the reward $R_{i}$ obtained from all flows through this agent, which can be expressed as the objective function:

(27) $$R_{i}=\gamma \sum_{t=1}^{\infty }r_{i}^{t}$$

The parameter γ∈[0,1) represents the discount factor of the reward function. Additionally, during the model training phase, an auxiliary state vector of length $N_{s}$ is appended for each traffic flow as input, wherein the nodes selected by the current traffic flow in the routing process are assigned a state of 1, while all other nodes are assigned a state of 0, in order to prevent the occurrence of routing loops.

### 4.3. Design of Resource Scheduling and Routing Algorithm Based on MAPPO

In the preceding sections, we have conducted a detailed design of node feature vector extraction, as well as the state, action, and reward functions of the DRL framework, based on the GraphSAGE model and multi-agent Markov processes. Within the design of the action space, the selection of routing nodes and bandwidth resource allocation are treated as discrete actions, whereas the allocation of computational and storage resources is considered as continuous actions. The MAPPO algorithm retains the “clipping” proximal update strategy of PPO, which mitigates policy mutation through constraints on policy updates, thereby ensuring stability throughout the training process and making it suitable for the frequently changing network states characteristic of the Mesh network scenario discussed here. Furthermore, due to the integration of optimization objectives for both discrete and continuous actions within the objective function, the MAPPO algorithm applies to mixed action spaces comprising both discrete and continuous actions. Consequently, this paper selects the MAPPO algorithm as the specific implementation for the DRL component, which will be elaborated upon in the following section.

**Figure 3:**
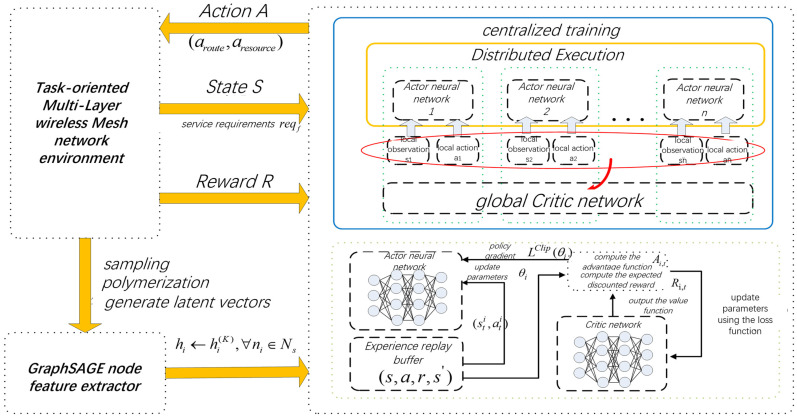
Architecture of the Service flow Routing and Resource Scheduling Algorithm Based on MAPPO.

As illustrated in Figure 3, an intelligent agent is deployed at each base station node, where the state of each agent at time slot t is represented as $s_{i}^{t}$. This state is composed of the node hidden vector $h_{i}$ Outputted by the GraphSAGE above and the current service flow demand. Subsequently, the action $a_{i}^{t}$ is derived from the policy function $\pi_{i}(a|s)$. The reward function $r_{i}^{t}$ is synthesized based on multiple QoS metrics, including latency, packet loss rate, transmission rate, and AI training accuracy, to characterize the contribution of the current action to the service utility function of the service flow. The DRL model employs an Actor-Critic neural network architecture to approximate the policy function, thereby generating the optimal input-output mapping relationship.

In a Markov Decision Process, agent i maps the input state $s_{i}^{t}$ to a probability distribution vector $\left\{p^{1},p^{2},\ldots ,p^{q}\right\}$ over a discrete action space through a parameterized policy function $\pi_{i}(a|s)$, where q represents the number of adjacent nodes at the node $n_{i}$. The routing algorithm selects the next-hop node corresponding to the maximum probability and performs resource allocation as the decision action $a_{i}^{t}$, subsequently generating an immediate reward $r_{i}^{t}$ upon the issuance of the routing decision. It is important to note that the resource allocation action is not included in the probability vector output by the agent during the algorithm’s implementation; otherwise, this would result in an excessively large action space. In fact, the input state space of the DRL model encompasses the hidden feature vectors of each node, thereby incorporating resource capabilities into the decision-making process. If the resources of a node can meet the service demands of a post-routing decision, they are allocated; otherwise, a penalty reward is issued.

In the distributed routing model, agents are required to collaborate to identify an optimal strategy $\pi_{\theta }(a|s)$ that maximizes the overall return of network routing decisions. Let the global objective function be denoted as R(θ), where θ represents the collection of neural network policy parameters for each agent. The gradient estimate for agent i during time slot *t* is expressed as:

(28) $$\nabla_{\theta_{i}}R(\theta_{i})=E_{t}[r^{t}(\theta_{i})\nabla_{\theta_{i}}\log\pi_{\theta_{i}}(a_{i}^{t}|s_{i}^{t})A_{i}^{t}]$$

where $r^{t}(\theta_{i})=\frac{\pi_{\theta_{i}}(a_{i}^{t}|s_{i}^{t})}{{\pi^{\prime }}_{\theta_{i}}(a_{i}^{t}|s_{i}^{t})}$ denotes the ratio of probabilities of generating the same policy using new and old strategies within time slot *t*. By manipulating the ratio of old to new strategies $r^{t}(\theta_{i})$ the effectiveness of the agent’s learning can be influenced. $A_{i}^{t}$ is the advantage function estimate. To prevent the update of $r^{t}(\theta_{i})$ from occurring too frequently, the MAPPO algorithm introduces a clipped objective function, constraining it within the interval (1−ε,1+ε) to limit the magnitude of policy changes during each update, where ε is the clipping threshold. Therefore, the objective function of the Actor network for agent i at time slot t is represented as:

(29) $$L(\theta_{i})=E_{t}[\min(r^{t}(\theta_{i})A_{i}^{t},clip(r^{t}(\theta_{i}),1-\varepsilon ,1+\varepsilon )A_{i}^{t})]$$

When the advantage function $A_{i}^{t}>0$, it indicates that the current action yields an expected return that exceeds that of the baseline policy. In this case, the probability weight of the action should be reinforced, while taking the minimum of $r^{t}(\theta_{i})$ and 1−ε to prevent over-optimization. Conversely, when the advantage function $A_{i}^{t}<0$, it is necessary to reduce the probability of selecting the current action, taking the maximum of $r^{t}(\theta_{i})$ and 1−ε to avert excessive policy shifts.

The advantage function can be estimated using the following formula:

(30) $$A_{i}^{t}=\delta_{t}+(\gamma \lambda )\delta_{t}+\ldots +{\left(\gamma \lambda \right)}^{T-t+1}\delta_{T-1}$$

In this context, $\delta_{t}$ represents the time difference error, defined as:

(31) $$\delta_{t}=r^{t}(\theta_{i})+\gamma V(s_{t+1})-V(s_{t})$$

In this process, the state-value function of the Critic network, denoted as $V(s_{t})$, is utilized to assess the quality of the overall policy of the network at the current moment. The Critic network calculates the temporal difference error by incorporating the state values $V(s_{t})$ and $V(s_{t+1})$, along with the reward $r^{t}(\theta_{i})$, and introduces a weighting parameter γ. Let the parameters of the Critic network be denoted as ω, to minimize the mean squared error of the value function.

(32) $$L(\omega )={\sum_{t=0}\left[r^{t}(\theta_{i})+\gamma V(s_{t+1})-V(s_{t})\right]}^{2}$$

Each agent utilizes the MAPPO algorithm to update policy parameters, and the specific process for routing and resource allocation decision-making is illustrated in Algorithm 2.
**Algorithm 2.** Distributed Routing Decision Algorithm Based on GraphSAGE-MAPPO.**Input:** the service flow arriving at agent i $f_{ij}=\{type_{f_{ij}},data_{f_{ij}},C_{f_{ij}}^{demand},req_{f_{ij}}\}$
Output: $\left(a_{route},a_{resource}\right)$, routing and resource allocation actions.1:initialize the network G, Actor network parameters θ, Critic network parameters ω, Experience replay buffer D.2:while $episode<N_{episodes}$3:    for step from 1 to T4:      if the current node is not the destination of the traffic flow.5:Obtain the initial feature vector of node $n_{i}$ $x_{n_{i}}=\{pos_{n_{i}},B_{n_{i}},C_{n_{i}},S_{n_{i}}\}$.6:Compute the hidden feature vector of the node using the algorithm in Algorithm 1. $h_{i}$, form the state space $s_{i}$ by incorporating the traffic flow $f_{ij}$7:Generate the next-hop probability distribution according to the policy function $\pi_{\theta_{i}}^{i}(a_{i},s_{i})$, select the optimal next hop, allocate resources, and compute the reward $R_{i}$ using Equation (26).8:Obtain the next state $s^{\prime }$, and store $\left(s,a,r,s^{\prime }\right)$ in the experience replay buffer D9:Sample experiences from the replay buffer and compute the advantage function $A_{i}^{t}$ according to Equation (30).10:Compute the loss function of the Actor network and update θ.11:Compute the loss function of the Critic network and update ω.12:    end if13:  end for14:end while15:Save the model.

The computational complexity of the proposed Distributed Routing Decision Algorithm is analyzed from two perspectives: the training phase and the inference phase. Let $N_{ep}$ be the number of episodes, T the steps per episode, B the batch size for updates, and d the feature dimension.

Time Complexity:

Training Phase: The algorithm runs for $N_{ep}$ episodes with T steps each. In each step, the computational cost consists of feature extraction, action generation, and network updates. Feature Extraction & Action: Calling the GraphSAGE module costs $O(N\cdot d^{2})$, and the Actor network forward pass costs $O(L\cdot d^{2})$, where L is the number of layers; Parameter Update: Updating the Actor and Critic networks involves processing a batch of size B. The backpropagation complexity is approximately $O(B\cdot d^{2})$; Total Training Time: $O(N_{ep}\cdot T\cdot (N\cdot d^{2}+B\cdot d^{2}))$. Since training is performed offline or in parallel, this cost is acceptable.

Inference Phase: During the mission, the UAV only executes the forward pass. For a single routing decision, the complexity is dominated by the local neighbor aggregation and the Actor network inference, which is $O(K\cdot s\cdot d+L\cdot d^{2})$. This complexity is independent of the global network size N and the iteration limit T, ensuring O(1) constant-time complexity per hop relative to the network scale, guaranteeing low latency for emergency communications.

Space Complexity:

The space consumption is primarily determined by the Experience Replay Buffer D and the neural network parameters; Replay Buffer: Storing transitions $\left(s,a,r,s^{\prime }\right)$ requires O(|D|·d), where D is the buffer capacity; Model Parameters: Storing weights for Actor (θ) and Critic (ω) networks requires $O(L\cdot d^{2})$. Total Space: $O(|D|\cdot d+L\cdot d^{2})$. This falls well within the memory constraints of modern UAV onboard processors.

## 5. Simulation Analysis

**Figure 4:**
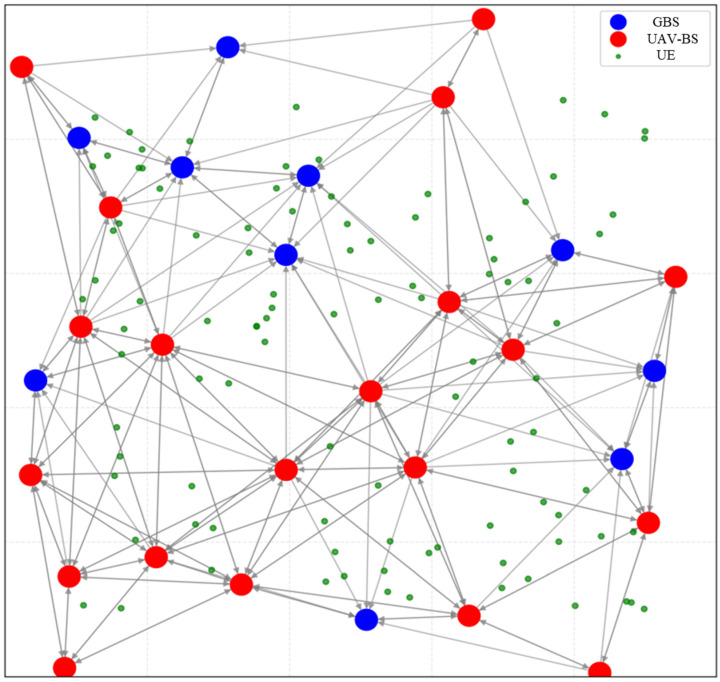
Distribution of Various Types of Base Stations and Users at Network Initialization.

This paper simulates the construction and deployment process of the GraphSAGE and MAPPO algorithm. The simulation experiments are conducted on a Windows 11 PC equipped with an AMD Ryzen 7 5800H CPU running at 3.20 GHz and 16 GB of RAM. Anaconda3-2020.02 is utilized to establish a Python virtual environment, and the deep learning framework is built using Python 3.7 in conjunction with TensorFlow 2.4.1. Initially, the physical network environment is constructed, considering the frequently changing characteristics of wireless mesh network topologies. The NetworkX library is employed to develop the underlying physical infrastructure network, and a directed graph is constructed to simulate real-world scenarios based on the coverage area of base station nodes. After achieving training stability, a small number of nodes and links are randomly added or removed every 500 training episodes to simulate topological changes. In each training episode, 400 tasks of various types are randomly generated, with the task size selectable from the set {3, 9, 15, 21, 27} Mbit. The relevant parameters of the pre-trained model for AI tasks are defined in Table 1, and the nodes for the transmission and reception of task data are selected randomly. The initial topology of the network is illustrated in Figure 4, while the settings for the physical network simulation parameters are provided in Table 2. The main parameter settings for the GraphSAGE-MAPPO algorithm model are outlined in Table 3.

Table 3 shows the key parameters of the GraphSAGE-MAPPO model, which are important for training the deep reinforcement learning agent. These are the parameters that govern issues such as network sampling, learning effort for actor and critic networks, exploration behavior and training setup.

This paper selects routing and resource allocation methods based on GraphSAGE-DQN [19], single-agent PPO [20] and the traditional Dijkstra algorithm as comparative algorithms, to validate the superior performance of the proposed algorithm under various traffic demand types and dynamic changes in network topology. The performance of the algorithms is assessed using metrics such as average delay, network throughput, packet loss rate, and training accuracy for AI services. Specifically, the GraphSAGE-DQN algorithm first utilizes a graph neural network to generate hidden feature vector representations of its nodes and subsequently employs a centralized DQN model to train routing for service flows with latency and rate requirements, making decisions by selecting entire paths from a pool of candidate paths for each service flow. The centralized PPO algorithm conducts hop-by-hop routing to maximize packet forwarding rates while minimizing latency. The Dijkstra algorithm greedily selects feasible shortest paths for each flow and allocates resources accordingly.

In the preceding text, we defined the reward function as shown in Equation (26). Considering the varying QoS requirements for different traffic types, we assigned the following weights to the metrics of delay, rate, and packet loss for rate-sensitive traffic flows: (0.25, 0.5, 0.25). For delay-sensitive traffic flows, the weights for delay, rate, and packet loss are (0.5, 0.25, 0.25). For packet loss-sensitive traffic flows, the corresponding weights are (0.25, 0.25, 0.5). For AI traffic flows, the weights assigned to delay, rate, packet loss, and training accuracy are (0.2, 0.2, 0.2, 0.4). Consequently, the average service utility of the traffic flows can be calculated using Equations (4) and (13), which subsequently allows for the computation of the overall reward value.

**Figure 5:**
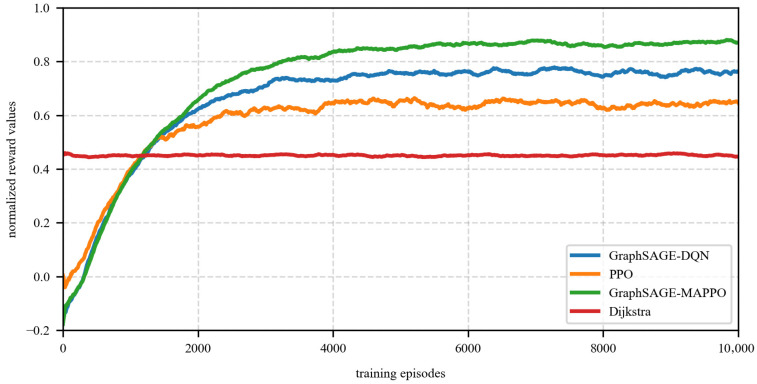
Normalized Reward Values.

The system normalization rewards under different algorithms are illustrated in Figure 5. To clearly visualize the convergence trends and mitigate high-frequency fluctuations caused by stochastic exploration, a moving average smoothing technique has been applied to the curves. From the figure, it can be observed that the reward values obtained from the flow routing and resource allocation algorithms based on the proposed method and GraphSAGE-DQN outperform those of other algorithms. Furthermore, when comparing the convergence speed, the PPO algorithm demonstrates superior performance relative to the proposed method and GraphSAGE-DQN. Specifically, the proposed algorithm converges after approximately 6000 iterations, while the PPO algorithm converges around 2000 iterations, and the GraphSAGE-DQN algorithm converges after approximately 4500 iterations. This indicates that the introduction of GNN for node feature extraction prior to DRL decision-making has a notable impact on algorithm complexity, and that multi-agent collaboration necessitates a greater number of training iterations to achieve a stable policy. The Dijkstra algorithm, as a deterministic shortest path algorithm, consistently pursues the shortest path for all reachable flows, with its policy remaining unchanged as the number of training iterations increases.

**Figure 6:**
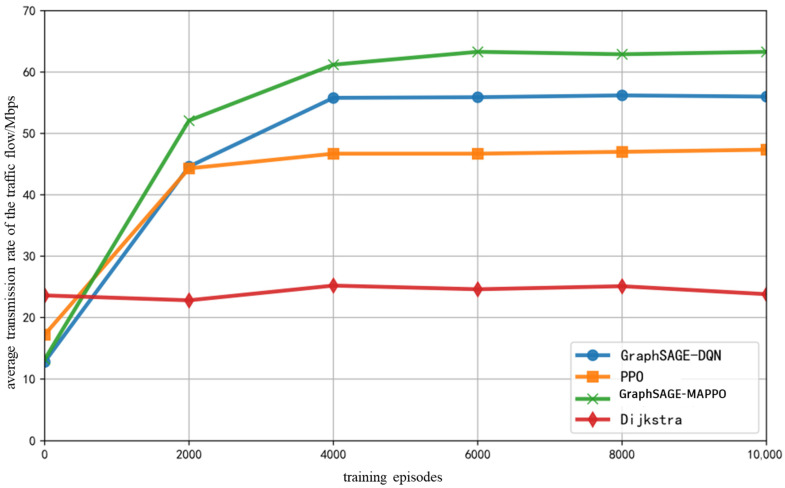
Average Transmission Rate of The Traffic Flow with Training Epochs.

As illustrated in Figure 6, during the process of random flow generation, the demand is set for high bandwidth, low latency, and low packet loss rate service flows, with the proportion of AI service flows being {0.4, 0.2, 0.2, 0.2}. As the number of training iterations increases, the average transmission rate of various service flows obtained by different intelligent algorithms initially grows rapidly before stabilizing. Although convergence is relatively slow, the algorithm proposed in this paper, along with the GraphSAGE-DQN algorithm, achieves a commendable average transmission rate for service flows, demonstrating the enhancement of traditional DRL agents’ decision-making capabilities through the prior updating of extracted network features, which allows for more than just local information perception. The PPO algorithm cannot perceive neighbor node features and relies on a single agent to learn the policy; consequently, it serves incoming traffic sequentially, rendering it unable to promptly adapt to changes in resource and link conditions within the environment, thus only achieving local optimality. The Dijkstra algorithm employs a fixed shortest-path strategy for scheduling various flows, resulting in low bandwidth resource utilization and a propensity for the emergence of “bottleneck links”.

**Figure 7:**
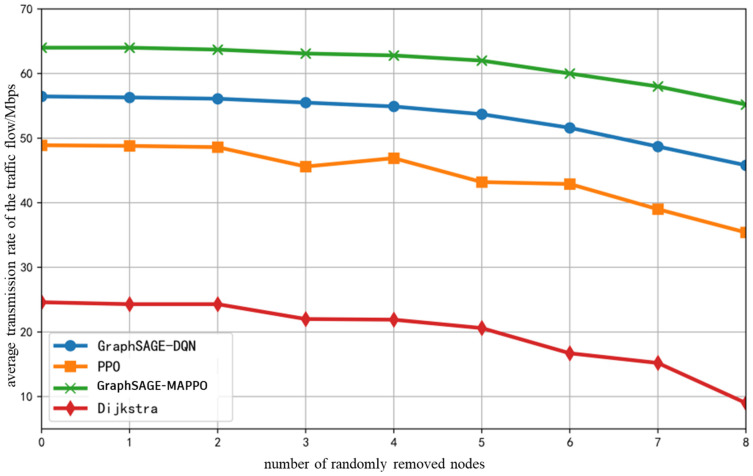
Average Transmission Rate of The Traffic Flow with the Number of Randomly Removed Nodes.

To validate the performance of the algorithm in dynamic environments, a fixed training iteration counts of 10,000 was established, with a total of 30 initial ground base stations and drones in the network. Following the specified proportions of various service flows, nodes and links were randomly removed after training stabilized around the 6000th iteration for all algorithms. The average end-to-end service flow rate, as depicted in Figure 7, varies with the number of randomly removed drones or ground base stations. Overall, as the number of randomly removed base stations increases, the average service flow rate obtained by all algorithms exhibits a downward trend. The proposed algorithm and the GraphSAGE-DQN algorithm demonstrate a relatively smaller and more stable decrease in average rate, reflecting a commendable adaptability to changes in network topology. Conversely, the PPO algorithm experiences fluctuations in performance due to alterations in node and link conditions following topology changes, necessitating retraining to explore new optimal strategies for convergence. The Dijkstra algorithm, when confronted with a significant number of node removals, suffers from insufficient bandwidth resource allocation at “central nodes,” resulting in a performance that is significantly influenced by the randomness of node removals, leading to the most considerable overall rate decline.

**Figure 8:**
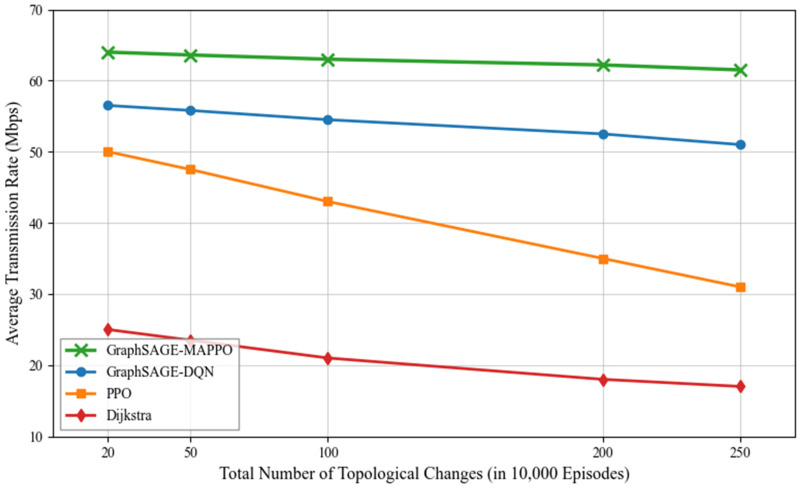
Average Transmission Rate of The Traffic Flow with the Frequency of Topological Changes.

To further rigorously validate the adaptability and generalization performance of the proposed algorithm in highly dynamic emergency scenarios, we conducted a sensitivity analysis regarding the frequency of topological changes. Figure 8 illustrates the average transmission rate of the traffic flow with respect to the varying topological update frequency. As the update frequency increases, the environment becomes increasingly dynamic, posing greater challenges to routing stability. It can be observed that the average transmission rates obtained by the baseline algorithms, particularly PPO and Dijkstra, exhibit a significant downward trend. The PPO algorithm experiences performance degradation because standard reinforcement learning methods struggle to re-converge and explore optimal strategies within shorter stable periods. Similarly, the Dijkstra algorithm suffers from frequent path invalidations caused by rapid link changes. Conversely, the proposed GraphSAGE-MAPPO algorithm demonstrates superior robustness. As shown in Figure 8, its performance curve remains relatively stable and maintains a high transmission rate even under the highest update frequency of 250 updates per 10,000 episodes. This demonstrates that the algorithm is insensitive to the frequency of topological changes. This robustness is attributed to the inductive learning capability of the GraphSAGE encoder, which learns generalizable aggregator functions rather than overfitting to specific network snapshots, thereby allowing the agent to rapidly adapt to new topologies without extensive retraining.

**Figure 9:**
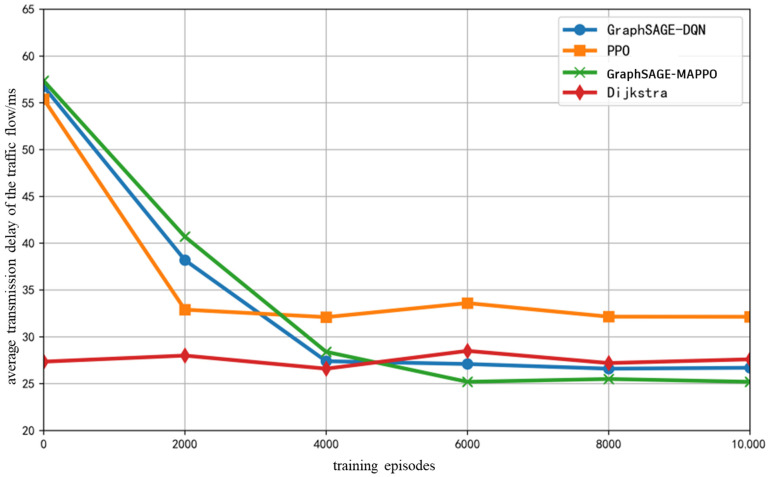
Average Transmission Delay of The Traffic Flow with Training Epochs.

As illustrated in Figure 9, with the demand for high bandwidth, low latency, and low packet loss rate service flows, as well as an AI service flow ratio of {0.2, 0.4, 0.2, 0.2}, the average transmission delay of various intelligent algorithm service flows initially decreases and then stabilizes as the number of training epochs varies. The GraphSAGE-DQN algorithm and the algorithm proposed in this paper exhibit relatively low average transmission delays once training stabilizes, as the inclusion of neighboring node features in the state input expands the agent’s observation space, thereby reducing unnecessary exploration of distant nodes. Furthermore, the DQN algorithm, by selecting entire paths directly from K shortest paths, mitigates certain deficiencies in the decision-making capabilities of a single agent. The Dijkstra algorithm, as a classical shortest path algorithm, achieves superior average end-to-end latency; however, due to the lack of scheduling strategies, when previously arrived flows greedily occupy resources on shorter paths, subsequent flows experience a higher hop count, thereby increasing latency.

**Figure 10:**
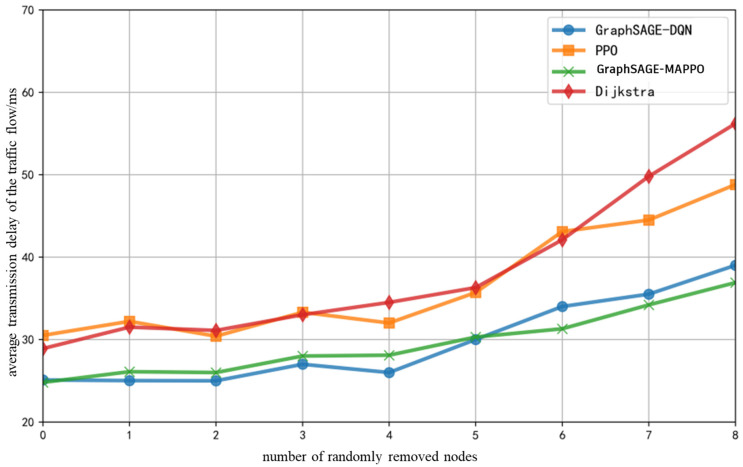
Average Transmission Delay of The Traffic Flow with the Number of Randomly Removed Nodes.

The fixed number of training iterations is set to 10,000, with the initial total number of ground base stations and drones in the network being 30. Under the previously specified proportions of various service flows, as the number of randomly removed drones or ground base stations increases, the variation in end-to-end average transmission delay of the service flows is illustrated in Figure 10. It can be observed that the overall average delay of all algorithms exhibits an increase; however, the proposed algorithm and the GraphSAGE-DQN algorithm show a relatively smaller increment, while the delay performance of the PPO algorithm experiences significant fluctuations due to re-convergence training following topology changes. The traditional Dijkstra algorithm, being greedy in its utilization of shortest path resources, results in a rapid increase in the hop count of subsequently arriving flows as the number of randomly removed nodes rises, leading to a substantial reduction in the availability of selectable resources.

**Figure 11:**
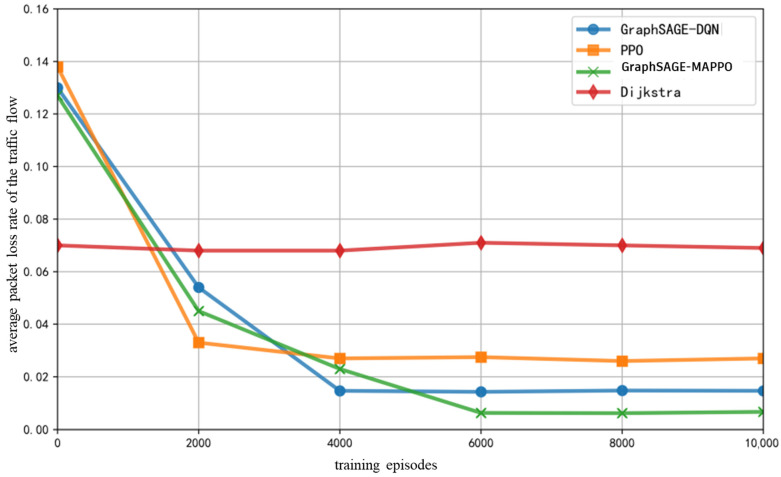
Average Packet Loss Rate of The Traffic Flow with Training Epochs.

As illustrated in Figure 11, with a bandwidth-intensive, low-latency, and low-packet-loss traffic flow ratio set to {0.2, 0.2, 0.4, 0.2}, the average packet loss rate of various intelligent algorithms initially decreases rapidly with increasing training epochs, followed by a gradual slowdown and eventual stabilization, indicating convergence. The proposed algorithm in this paper, along with the GraphSAGE-DQN algorithm, exhibits a relatively low packet loss rate after achieving training stability. This is attributed to the relationship between packet loss rate and link transmission quality. In the GraphSAGE algorithm, each node’s features aggregate the characteristics of neighboring nodes and connected links, which include average packet loss rate information gathered from the experience buffer. This enables the DRL model to avoid congested links and resource-deficient nodes during exploration. Conversely, the Dijkstra algorithm’s greedy selection of the shortest path results in congestion on certain links, leading to the highest overall packet loss rate.

**Figure 12:**
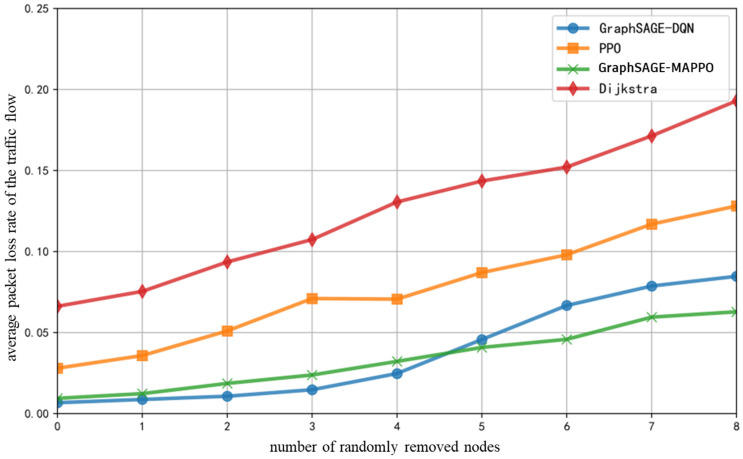
Average Packet Loss Rate of The Traffic Flow with the Number of Randomly Removed Nodes.

The fixed number of training iterations is set to 10,000, with the initial total number of ground base stations and drones in the network being 30. Under the previously specified proportions of various service flows, as the number of randomly removed drones or ground base stations increases, the variation in the end-to-end average packet loss rate of the service flows is illustrated in Figure 12. The average packet loss rate of all algorithms increases with the removal of network nodes. Compared to benchmark algorithms, the proposed algorithm in this study exhibits the least increase in packet loss rate. This is attributed to the significant changes in network topology when a larger number of nodes are removed. The proposed algorithm extracts node features and integrates the link packet loss rate metrics, allowing the DRL decision-making process to merely require the re-extraction of hidden features to identify the next-hop nodes and links with lower real-time packet loss rates, effectively avoiding congested links.

**Figure 13:**
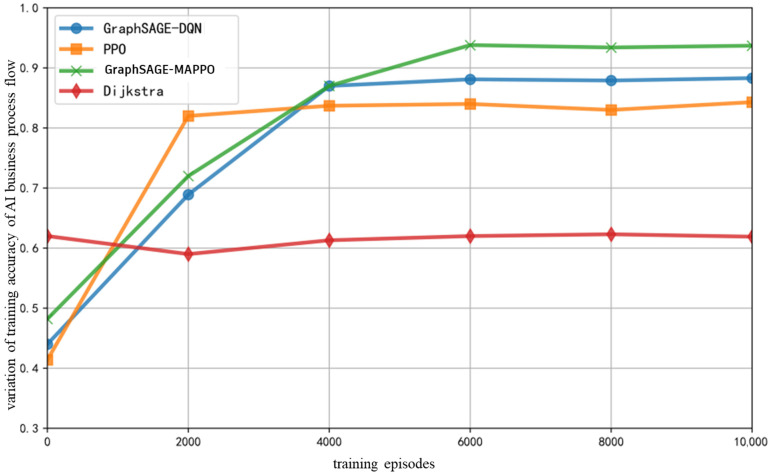
Variation of Training Accuracy of AI Service Process Flow with Respect to Training Epochs.

As illustrated in Figure 13, for the AI service flow, the training accuracy of various intelligent algorithms gradually increases and reaches a stable value with the increase in training epochs. The training accuracy of the AI service flow is contingent upon the compression of the training model and the quality of the samples, where the model compression is determined by the resource capabilities of the nodes, and the sample quality is influenced by the transmission quality of hop-by-hop links. The algorithms proposed in this paper, along with the GraphSAGE-DQN algorithm, extract node feature vectors that encompass link packet loss rates and node resource information. This effectively reduces the search space during DRL decision-making, avoids low-quality links, and enhances data quality, while routing tasks to resource-abundant Mesh nodes and employing a full model to improve accuracy. In contrast, the single-agent PPO algorithm lacks an effective perception of the surrounding environment of each node, leading to a local optimum. The Dijkstra algorithm is deficient in scheduling strategies, resulting in low data quality over congested links; the greedy selection of central nodes imposes resource constraints, and the adoption of low-precision models further diminishes overall accuracy.

Finally, regarding practical applicability, the proposed GraphSAGE-MAPPO framework inherently supports scalability through its architectural design. Unlike traditional centralized algorithms that suffer from computational bottlenecks as the network size (N) grows, the inductive nature of GraphSAGE utilizes neighbor sampling, decoupling the inference complexity from the total number of nodes. Furthermore, the decentralized execution paradigm allows each UAV to make routing decisions based solely on local observations, effectively preventing communication congestion and control signaling overhead even under high traffic loads or in large-scale dense networks. This ensures the solution remains viable for extensive emergency deployment.

## 6. Conclusions

In response to the limitations of existing deep reinforcement learning-based intelligent routing and resource allocation algorithms in adapting to the dynamic changes in network structures and demands of wireless networks, this paper proposes an intelligent routing algorithm, GraphSAGE-MAPPO, which integrates GNN and DRL. During the training process, GraphSAGE is utilized to extract aggregated features of the nodes and links, which are then combined with the status of service flows to serve as the state input for the subsequent DRL algorithm. The model is trained to make hop-by-hop routing decisions and allocate resources efficiently. Experimental results demonstrate that, compared to the GraphSAGE-DQN algorithm and the PPO and Dijkstra algorithms that do not employ GNN for network feature extraction, the proposed algorithm exhibits superior performance in terms of average latency, packet loss rate, network throughput, and training accuracy for AI services. Furthermore, it demonstrates robust generalization capabilities in the event of network node failures, with the trained model effectively transferring to dynamically changing scenarios.

## Figures and Tables

**Table 1:** Performance Parameters of Pre-trained DNN Models.

Model Scale	Model Accuracy	Computational Demand/MHz	Model Size/MB
z=0 (lightweight model)	[0.929, 0.954]	[20, 30]	[3.5, 5.5]
z=1 (full-scale model)	[0.973, 0.992]	[80, 120]	[9, 12]

**Table 2:** Parameters for Physical Network Simulation.

Physical Network Environment Parameters	Parameter Value
simulation area size m^2^	1000 × 1000
number of ground base stations	[5, 10]
GBS coverage radius (m)	200
number of UAV-BS	[15, 20]
UAV-BS coverage radius (m)	400
base station allocatable channel bandwidth (MHz)	20
computing resources of various base stations (GHz)	[1, 2]
base station storage resources (MB)	[500, 800]
link transmission delay (ms)	[4, 10]
link packet loss rate (%)	[0.01, 10]
tolerable delay of delay-sensitive task flows (ms)	[30, 50]
tolerable rate threshold of rate-demanding task flows (Mbps)	[30, 50]
packet loss tolerance threshold of loss-sensitive task flows (%)	[1, 5]
training accuracy tolerance of AI tasks (%)	[80, 90]

**Table 3:** Key Parameters of the GraphSAGE-MAPPO Model.

Trainable Model Parameters	Parameter Value
GraphSAGE sampling depth K	2
GraphSAGE per-layer node sampling number s	5
Actor network learning rate	0.0003
Critic network learning rate	0.001
reward discount factor	0.99
initial value of the exploration rate	1.0
final value of the exploration rate	0.01
rate of decay for the exploration rate	0.995
PPO clip parameter ϵ	0.2
replay buffer size	5000
batch size of randomly sampled training data	64
training episodes	10,000
max steps in each episode	50

## Data Availability

All data generated or analyzed during this study are included in this published article.

## Abbreviations

The following abbreviations are used in this manuscript:

AI	Artificial Intelligence
MAPPO	Multi-Agent Proximal Policy Optimization
GNN	Graph Neural Networks
DRL	Deep Reinforcement Learning
URLLC	Ultra-Reliable Low Latency Communication
DQN	Deep Q-Networks
GMAPPO	GNN-based Multi-Agent Proximal Policy Optimization
UAV	unmanned aerial vehicle
SNR	signal-to-noise ratio
AWGN	additive white Gaussian noise
GNN–MADRL	Graph Neural Network based Multi-Agent Deep Reinforcement Learning
